# Decellularization Strategies for Regenerating Cardiac and Skeletal Muscle Tissues

**DOI:** 10.3389/fbioe.2022.831300

**Published:** 2022-02-28

**Authors:** Yong How Tan, Haylie R. Helms, Karina H. Nakayama

**Affiliations:** Department of Biomedical Engineering, Oregon Health and Science University, Portland, OR, United States

**Keywords:** decellularized muscle, decellularized heart, dECM, decellularized extracellular matrix, skeletal muscle engineering, cardiac engineered tissue, ECM, extracellular matrix

## Abstract

Cardiovascular disease is the leading cause of death worldwide and is associated with approximately 17.9 million deaths each year. Musculoskeletal conditions affect more than 1.71 billion people globally and are the leading cause of disability. These two areas represent a massive global health burden that is perpetuated by a lack of functionally restorative treatment options. The fields of regenerative medicine and tissue engineering offer great promise for the development of therapies to repair damaged or diseased tissues. Decellularized tissues and extracellular matrices are cornerstones of regenerative biomaterials and have been used clinically for decades and many have received FDA approval. In this review, we first discuss and compare methods used to produce decellularized tissues and ECMs from cardiac and skeletal muscle. We take a focused look at how different biophysical properties such as spatial topography, extracellular matrix composition, and mechanical characteristics influence cell behavior and function in the context of regenerative medicine. Lastly, we describe emerging research and forecast the future high impact applications of decellularized cardiac and skeletal muscle that will drive novel and effective regenerative therapies.

## 1 Introduction

Cardiovascular disease and musculoskeletal conditions together represent a major global health burden. According to the World Health Organization (WHO), cardiovascular disease is the leading cause of death worldwide and accounts for an estimated 17.9 million deaths annually (World Health Organization, 2021a). Separately, an astonishing 1.71 billion global citizens are affected by musculoskeletal conditions and is the leading cause of disability in 160 countries (World Health Organization, 2021b). Combined, these two health crises are responsible for a global annual economic burden of approximately $1.8 trillion (Bloom et al., 2011; Yelin et al., 2021). These statistics are projected to only worsen by 2030 unless a major shift in treatment options becomes widely available. Regenerative medicine reconceptualizes the approach to this challenge with technologies that potentiate cures over treatments. Through the modulation of the body’s response to injury and disease using bioactive materials, the potential for full restoration of function is on the horizon.

Decellularized tissues and extracellular matrices (ECM) are the heavyweights of regenerative biomaterials with decades of clinical use and FDA approvals. These materials possess potent regenerative properties and have the potential to repair and restore function to a wide range of injured and diseased tissues including cardiac and skeletal muscle. In this review, the application of decellularized ECM (dECM) in the cellular modulation and regeneration of cardiac and skeletal muscle is described. Specifically, the most widely used decellularization protocols for generating dECM from these two tissues are compared. Herein, we describe how the biophysical properties of composition, topographical and spatial patterning, and biomechanical force transduction are impacted by the decellularization protocol as well as characterize the modulatory role that these properties play in guiding cellular and host interactions *in vitro* and *in vivo*.

## 2 Methods of Decellularization

**FIGURE 1:**
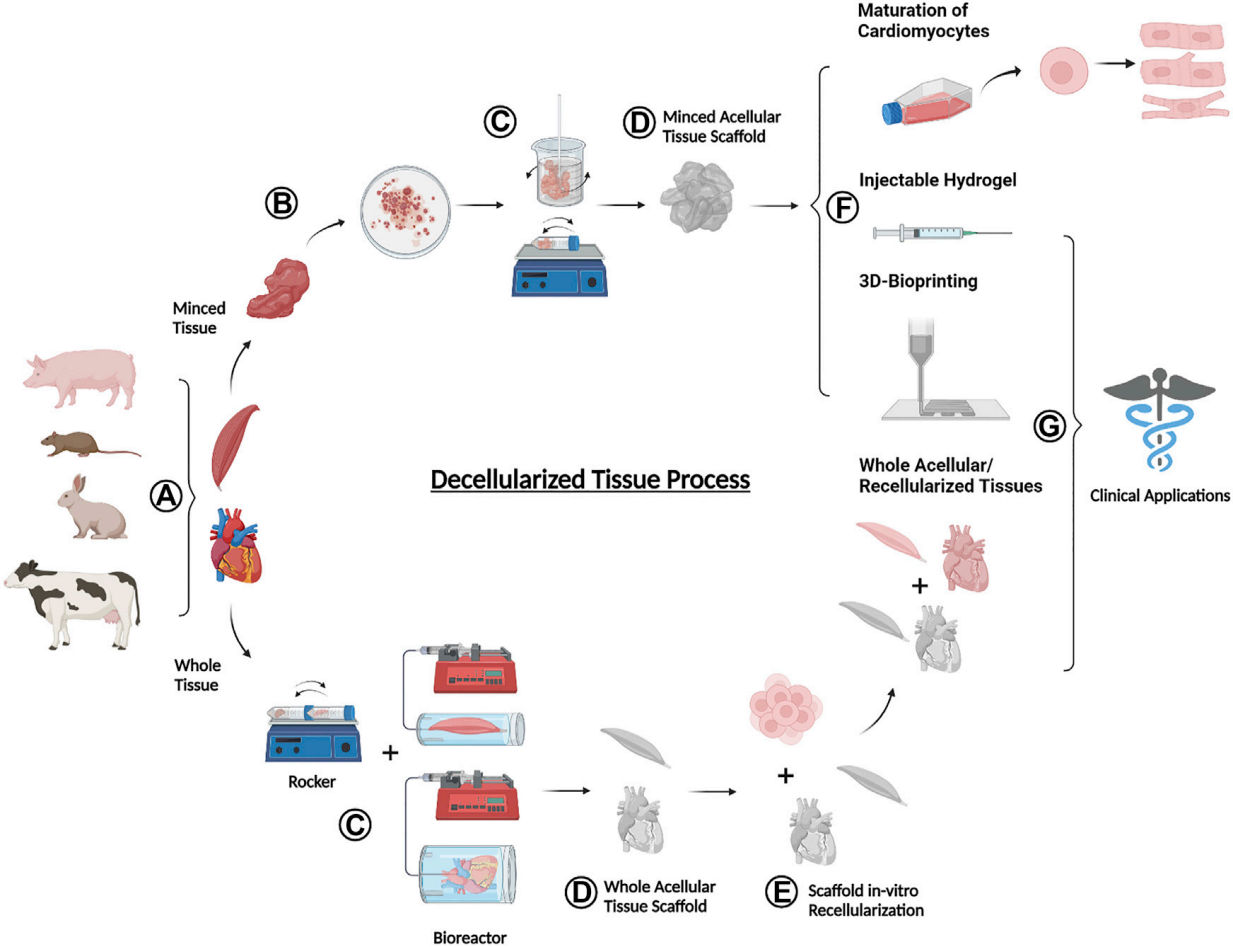
Schematic overview of the donor-to-application lifecycle of decellularized tissues **(A)** Tissues are harvested from an animal donor **(B)** Whole tissues are processed with mechanical agitation to break down into smaller minced tissues **(C)** Whole or minced fresh tissues are exposed to a series of decellularizing agents and procedures to remove cellular materials **(D)** The decellularization end products are acellular tissues containing various ECM components **(E)** Whole acellular tissues may be seeded with cells *in vitro* to test for cell viability **(F)** A variety of dECM forms are used for the maturation of cardiomyocytes, production of injectable hydrogels, and formulation into bioink for 3D bioprinting **(G)** Selected forms of decellularized tissues including injectable hydrogels, 3D bioprinted materials, and whole acellular and recellularized tissues can be used for translational pre-clinical and clinical applications. Figure created using BioRender.com.

The global process of decellularization involves a series of steps that take the tissue from donor to regenerative application (Figure 1). This is done by harvesting tissues from a donor animal (Figure 1A), followed by the application of a series of reagent and wash solutions that lyse cells and remove nuclear materials (Figure 1B,C) to leave behind a structural matrix made up of ECM proteins (Figure 1D). The resulting dECM can then be used as a cell culture tool, solubilized into bioink for 3D printing, or cultured with cells to be transplanted as a regenerative therapeutic into an animal injury model (Figure 1E–G).

Historically, tissues have been decellularized primarily via three methods that are based in chemical, physical, and/or enzymatic disruption of the cell-matrix interface. Chemical decellularization utilizes detergents, alcohols, or pH changes to lyse cells by disrupting the cell membrane (Gilpin and Yang, 2017; KC et al., 2019). Methods of physical decellularization include freeze-thaw cycles, agitation, mechanical manipulation, or pressure to forcibly remove cells (Gilpin and Yang, 2017; KC et al., 2019). Lastly, enzymatic decellularization breaks down the protein interactions through the use of nucleases, collagenases, and proteases and/or chelating agents such as ethylenediamine tetraacetic acid (EDTA) or ethylene glycol tetraacetic acid (EGTA) (Gilpin and Yang, 2017; KC et al., 2019). Along with each decellularization approach are associated advantages and disadvantages which must be carefully considered for the end application.

Modern decellularization strategies often involve a balance between effective and efficient removal of cellular debris with the loss of key proteins and matrix components. For example, sodium dodecyl sulfate (SDS) is a chemical decellularization agent that is highly effective in lysing cells but can alter the microstructure of ECM and is cytotoxic if not thoroughly removed (Gilpin and Yang, 2017). Another widely used detergent, Triton X-100, generally preserves the ECM composition and biomechanics but is less effective in the complete clearing of nuclear material (Ott et al., 2008; Merna et al., 2013; Gilpin and Yang, 2017). Similarly, physical decellularization has a high retention and structural preservation of ECM proteins and mechanical properties but is more likely to leave remnant DNA or DNA fragments behind (Gilpin and Yang, 2017). Enzymatic decellularization involves potent digestive enzymes that are efficient at breaking down and removing cellular debris but has been found to decrease glycosaminoglycan (GAG) content and degrade crosslinking proteins leading to altered mechanical properties (Gilpin and Yang, 2017). There is no universal reagent or protocol, but instead the selection of the most appropriate decellularization approach heavily depends on the final application and will dictate the hierarchy of properties to retain. The choice is further complicated by the compositional variations between tissue types, specifically differences in anatomical structure, function, and age. In this section we discuss the range of methods used to decellularize two kinds of muscle tissues, cardiac muscle and skeletal muscle.

### 2.1 Decellularization Methods for Cardiac Tissue

In 2008, Doris Taylor’s group reported the landmark decellularization of a whole heart and simultaneously catapulted an emerging field to the forefront of tissue engineering and regenerative medicine (Ott et al., 2008). Their chemical decellularization approach utilized perfusion of 1% SDS and 1% Triton X-100, with deionized water and 1X PBS washes. This dynamic protocol proved effective in removing native cellular material (residual DNA <4%) while preserving GAG content and fiber composition. The resulting dECM matrix supported the culture of neonatal cardiac cells, without any noted complications from residual reagents. Other whole heart decellularization protocols have built upon the concepts initially established by Ott et al. (Ott et al., 2008) and even this group has since modified their protocol to incorporate osmotic shock and anatomical inversion during decellularization (Lee et al., 2017) (Table 1). Nevertheless, since 2008, this protocol has continued to serve as a foundation for tissue decellularization strategies and a major catalyst for innovation.

**TABLE 1:** Cardiac decellularization protocols.

Application	Species	Tissue form	Decellularization treatments	Study findings	Citation
Decell Methods	Porcine	Whole	Inversion +45°; NaCl; SDS	Improved perfusion efficiency; better DNA clearance, retention of ECM and heart shape	Lee et al. (2017)
Decell Methods	Porcine	Whole	Freeze/thaw; SDC; Triton; PAA	Extensive decellularization while retaining architecture and angiogenic growth factors	Methe et al. (2014)
Decell Methods	Porcine	Whole	1. Freeze/thaw; Trypsin 2. Freeze/thaw; Triton	Multiphoton microscopy and image correlation spectroscopy can predict mechanical properties of dECM	Merna et al. (2013)
Decell Methods	Rat	Whole	Freeze/thaw; Trypsin; SDS; Triton; DCA; PAA + EtOH	Little residual nuclear material; minimizes damage to proteins; retention of heart shape	Ozlu et al., 2019
Decell/Recell Methods	Porcine	Whole	Freeze/thaw; Trypsin; Triton; DCA; PAA + EtOH	Significantly reduced decellularization time to 10 h without damaging the ECM	Wainwright et al. (2010)
Decell/Recell Methods	Rat	Whole	SDS; Triton	Preserved ECM characteristics; promoted hESC-CM attachment, maturation, and electrical activity	Hochman-Mendez et al. (2020)
Decell/Recell Methods	Rat	Whole	Heparin, Adenosine; SDS; Triton	ECM preserved and vasculature perfusable; cardiac and EC recellularization; stimulation produced myocardial contractions	Ott et al. (2008)
Decell/Recell Methods	Mouse	Whole	Freeze/thaw; Trypsin; SDS; Triton; DCA; PAA + EtOH	Cardiac progenitors grown on dECM migrate, proliferate, and differentiate into CMs, SMC, and ECs	Lu et al. (2013)
Recell Methods	Rat	Whole	SDS; Triton	Reendothelialization best with both venous and arterial delivery	Robertson et al. (2014)
Cell Culture Additive	Human	Minced	NaCl; SDS	Atrial dECM in hiPSC-CM differentiation drives atrial cardiomyocyte subtype specification	Mesquita et al. (2021)
Cell Culture Additive	Porcine	Minced	SDS; Triton	Fetal ECM increased neonatal CM attachment and proliferation compared to PLL, adult ECM, or neonatal ECM	Williams et al. (2014)
Cell Culture Additive	Bovine	Minced	Freeze/thaw; SDS; Triton	3D ECM enhances iPSC-CM maturation compared to 2D culture	Fong et al. (2016)
Injectable Therapeutic	Porcine	Minced	SDS; Triton	Injection of solubilized fetal ECM decreased fibrosis and improved cardiac function post MI	Wang, X. et al., 2020
Injectable Therapeutic	Mouse	Minced	Freeze/thaw; RBC lysis; DNase I + RNase	Neonatal ECM decreased fibrosis, promoted angiogenesis, and increased cardiac function post MI; adult ECM did not have the same regenerative effects	Wang, Z. et al., 2019
Hydrogel	Porcine	Minced	SDS; Triton; PAA + EtOH	Developed a hydrogel with tunable mechanical and electrical properties; hydrogel improved hiPSC-CM maturation	Tsui et al. (2021)
Hydrogel	Bovine	Minced	SDS; Triton	Developed an electroactive, *in situ* forming, ECM hydrogel	Mousavi et al. (2021)
Hydrogel/Bioink	Human	Minced	Freeze/Thaw; SDS; Triton	GelMA-MeHA-ECM bioink improved mechanical properties; created an *in vitro* MI model with the bioink	Basara et al. (2021)
Hydrogel/Bioink	Porcine	Minced	SDS	Human cardiac progenitor cells in GelMA-ECM bioink had >75% viability and increased cardiogenic gene expression	Bejleri et al. (2018)

Optimization of cardiac decellularization has been guided by the dual goal of ensuring removal of cellular materials and preservation of ECM components. These aims motivated the next generation of decellularization strategies that sought to reduce exposure to harsh reagents through decreased decellularization times. Wainwright et al. was able to significantly reduce total decellularization time to 10 h, compared to the conventional 5–10 days, by using a series of hypertonic, hypotonic, enzymatic, acid, and detergent solutions without damaging the ECM (Wainwright et al., 2010). This time was reduced even further to 2 h and 15 min using a similar mixed method approach (Lu et al., 2013). However, there is no consensus on which component of a decellularization protocol is most critical and method selection is often based on the desired end application properties. In 2011, Akhyari et al. sought to find the best whole heart decellularization protocol in terms of cellular removal, residual DNA, retention of structural proteins, and biocompatibility (Akhyari et al., 2011). Following a comprehensive comparison of four different methods, using a fully automated system, it was concluded that no single approach was superior to the others. While some protocols were more effective at removal of cellular materials, others better preserved the native dECM proteins. Each protocol had a compromise of strengths and limitations and serves as another example to underscore the need to carefully select the appropriate decellularization method based on the final desired ECM properties.

In recent years, the cardiac field has seen a pivot away from using whole tissue matrices for tissue engineered and regenerative therapeutics towards dECM that is ground or solubilized with primary focus on answering *in vitro* biological questions. For these applications, the native heart tissue is minced, thereby increasing surface area, and significantly speeding up the decellularization process. The use of minced tissue also increases research accessibility by eliminating the need for specialized perfusion bioreactors to keep the whole heart intact, and instead utilizes basic tools for decellularization that are available in most research labs (stir bars/orbital shakers). Similar to whole heart decellularization, the minced tissue approach often uses detergents such as 1% SDS and 1% Triton X-100.

The properties linked to tissue origin such as species source and donor age also demonstrate differential responses to different decellularization protocols. An example of the influence of tissue source is evidenced by a study in which a protocol that had been optimized to decellularized fresh porcine hearts was not effective in the complete decellularization of the same sized cadaveric human hearts (Johnson et al., 2014). Cadaveric hearts required much longer SDS treatment in addition to isopropyl alcohol and a DNase/RNase solution containing HCl, MgCl, CaCl, and NaCl at pH 7.4. Even still, there was significant patient-to-patient variability, which increased the difficulty in processing the human tissues. These differences could possibly be explained by the variability in age amongst the cadaveric hearts as well as the difference in age between the human and porcine hearts. Age increases cardiac tissue stiffening through fibrosis and increased ECM cross-linking (Johnson et al., 2014). Similarly, fetal rat and porcine hearts required a more diluted concentration of SDS and Triton X-100 compared to their adult counterparts (Williams et al., 2014; Fong et al., 2016; Wang et al., 2020; Wang et al., 2021). However, there are rare instances in which a protocol has been developed that works reasonably well for tissues of disparate ages. Silva et al. successfully developed a protocol that enabled the parallel decellularization of both fetal and adult rat hearts using freeze-thaw, physical agitation, hypotonic buffer (Tris HCl + EDTA), 0.2% SDS, and DNase (Silva et al., 2016). In summary, there are many variables that influence the effectiveness of a cardiac decellularization protocol that heavily depend on the tissue species, age, and final end application.

### 2.2 Decellularization Methods for Skeletal Tissue

Over the course of the last decade, an increasing number of different protocols have been developed and modified for skeletal muscle decellularization (Table 2). When the field was relatively young, the most common decellularization agent was trypsin, which was often used in combination with detergents such as SDS, Triton X-100, and Sodium Deoxycholate (SDC). The use of trypsin eventually declined when the destructive properties of this enzyme became well-known (Miersch et al., 2018; Sharma et al., 2019). While a harsh decellularization treatment is highly efficient at removing cellular debris and DNA from the tissue, it can also remove essential ECM proteins and proteoglycans. The preservation of the ECM is especially relevant for skeletal muscle, which, based on a proteomic analysis of 21 different organs, has over a 10-fold greater number of unique proteins compared to all other organs (Raffa et al., 2020). The ECM serves as an important driver of cell-fate and tissue regeneration in the healing response of skeletal muscle following injury (Herrera et al., 2018; Csapo et al., 2020; Nicolas et al., 2020). Therefore, like cardiac decellularization protocols, methods for obtaining decellularized skeletal muscle ECM require a balance of removing cellular material while best conserving the biophysical components and integrity of the complex skeletal muscle matrix (Grisales et al., 2021).

**TABLE 2:** Skeletal muscle decellularization protocols.

Application	Species	Tissue Form	Decellularization Treatments	Study Findings	Citation
Comp/ Topo/ Mech	Porcine	Minced	SDS + Triton	dECM bioink improved muscle regeneration when compared to collagen bioink.	Choi et al. (2016)
Comp/ Mech	Rat	Whole	Free/thaw + Trypsin + Triton	*In vivo* transplantation of muscle derived ECM showed a large fibrotic mass production 6 months post-surgery.	Corona et al. (2013)
Comp/ Mech	Rabbit	Whole	LatB + KCl/KI	Xenotransplant scaffold showed low immunogenic response *in vitro* and *in vivo*, suggesting immunosuppressive and anti-inflammatory effects.	Fishman et al. (2013)
Comp/ Topo	Porcine	Minced	SDS Trypsin + Triton + SDC	Exploitation of PDMS elastic property allowed anisotropic reorganization of 3D skeletal muscle dECM; construct supports de novo muscle regeneration.	Jin et al. (2021a)
Comp/ Mech	Rat	Whole	SDS	dECM and minced muscle scaffolds improved recovery in VML injury animal model.	Kasukonis et al. (2016a)
Comp/ Mech	Rat	Whole	SDS	Developed an infusion bioreactor for skeletal muscle decellularization.	Kasukonis et al. (2016b)
Comp/ Mech	Rabbit	Minced	SDS + Triton	Development of cell-free scaffold comprised of decellularized skeletal muscle and IGF-1 promotes tissue regeneration in vivo and in situ.	Lee et al. (2020)
Comp	Human	Minced	LatB + KCl/KI SDC SDS	Harsher SDS treatment shown to decellularize human skeletal muscle better than less harsh LatB + KCl/KI and SDC treatments.	Naik et al. (2020)
Comp/Topo	Bovine	Minced	Freeze-thaw + Triton + NH4OH	Electrospun PCL/dECM scaffold supported myogenesis *in vitro*; scaffold implanted in murine VML *in vivo* model showed increased anti-inflammatory activity and myofiber regeneration.	Patel et al. (2020)
Comp/Mech	Chicken	Whole	LatB + KCl/KI Triton Triton + SDS	LatB + KCl/KI protocol was most efficient for decellularization compared to competing methods, reducing DNA, myosin and actin content significantly.	Reyna et al. (2020)
Comp/ Mech	Bovine	Minced	Freeze/thaw + Triton + NH4OH	In vitro studies of dECM gelloid supported MSC survival, expansion, trophic factor secretion, immunomodulation, and myogenic protein expression.	Talovic et al. (2019)
Comp/ Mech	Rat	Whole	LatB + KCl/KI SDC SDS	Implanted dECM from three different protocols in immune competent model of VML generated functional muscle, vasculature, nervous fibers.	Urciuolo et al. (2018)
Comp	Dog	Whole	Trypsin + SDC + Triton	Comparing decellularized small intestine and skeletal muscle regenerative outcomes shows no difference after prolonged period.	Wolf et al. (2012)
Comp	Porcine	Whole	Trypsin + SDS + Triton	*In vivo* implantation of dECM in partial thickness abdominal wall defects in rats showed neovascularization, myogenesis, and recellularization.	Zhang et al. (2016)
Topo/ Comp/ Mech	Porcine	Minced	SDS + Triton	dECM coating significantly improved myotube and myogenic differentiation compared with collagen/non-coated surfaces.	Choi et al. (2018)
Topo/ Comp/ Mech	Porcine	Minced	SDS + Triton	The 3D printed muscle constructs exhibited high cell viability without hypoxia; enhanced de novo muscle formation in a VML rat model.	Choi et al. (2019)
Topo/ Comp	Porcine	Minced	SDS	Thermally drawn PCL/dECM mix implanted in VML, *a* model showed induced de novo muscle regeneration.	Jin et al. (2021b)
Topo/ Comp/ Mech	Porcine	Minced	SDS + Triton	In vitro cellular responses showed successful recellularization on 3D-printed dECM-based structure.	Kim et al. (2020)
Topo/ Comp/ Mech	Porcine	Minced	SDS + Triton	Photo-crosslinkable, dECM/PVA hydrogel, 3D-printed self-aligned skeletal muscle construct showed rapid restoration of muscle function.	Lee et al. (2021)
Topo/ Comp/ Mech	Bovine	Minced	Freeze-thaw + Triton + NH4OH	Aligned electrospun dECM/PCL scaffolds supported satellite cell growth, myogenic protein expression and myokine production.	Patel et al. (2019)
Topo	Rabbit	Minced	Trypsin + Triton	C2C12 myoblasts recellularization affected by degree of crosslinking (stiffness) and alignment (topography) of electrospun dECM scaffold.	Smoak et al. (2021)

There exists a wide variety of approaches for obtaining decellularized skeletal muscle, which range from the more aggressive enzymatic treatments to the use of comparatively mild depolymerizing agents such as Latrunculin B (LatB). Detergent-based methods have historically dominated the field of skeletal muscle decellularization. The most widely used detergents such as SDS, Triton X-100, and SDC are used for their surfactant properties which disrupt the lipid bilayer of cell membranes to facilitate cell lysis (Lichtenberg et al., 2013). More specifically, SDS acts by solubilizing the cytoplasmic and nuclear membranes, while both Triton X-100 and SDC work by targeting the lipid-protein and lipid-lipid interactions of cells (Reyna et al., 2020). The mechanism of action of these different reagents can dictate the efficiency of cellular removal.

A treatment of 1% SDS has been used in numerous studies as the main decellularization agent for minced muscle and was commonly reported to remove approximately 98% total DNA (Choi et al., 2016; Kim et al., 2020; Lee et al., 2020; Jin et al., 2021a; Jin et al., 2021b). In contrast, groups that used non-SDS agents such as SDC and ammonium hydroxide reported less efficient cellular removal with a range of 50–80% total DNA (Piccoli et al., 2017; Patel et al., 2019; Smoak et al., 2019; Talovic et al., 2019; Jin et al., 2021a). Removal of cellular material and DNA content is critical in the downstream application of decellularized tissues to avoid complications triggered by the host immune response. Several residual components can lead to the activation of an immune response following allo- and xenotransplantation including any remaining DNA fragments, human leukocyte antigens (HLA), and major histocompatibility complex (MHC) molecules (Quake, 2011; Nakamura et al., 2019). However, while detergent-based methods are effective in clearing the tissue of cellular debris, these reagents may also remove key bioactive ECM proteins and proteoglycans (Gilbert et al., 2009; Crapo et al., 2011; Soto-Gutierrez et al., 2011). Therefore, skeletal muscle decellularization protocols must balance the removal of cellular materials with maintaining any essential cell signaling components contained within the ECM.

To mitigate the disruptive properties of detergent-based methods, gentler decellularization approaches have been developed for skeletal muscle. Utilizing a natural toxin produced by aquatic sponges (Spector et al., 1983), Gillies et al. demonstrated a decellularization procedure using LatB together with hyper- and hypotonic salt solutions (Gillies et al., 2011). The LatB treatment depolymerized the actin-myosin filaments of whole skeletal muscle structures in mice, while actively preserving the ECM components including GAGs and collagen (Gillies et al., 2011). Because this protocol is also effective at removing DNA (95% removal, 2.92 ± 0.14 mg/mg dry weight in native muscle to 0.12 ± 0.01 mg/mg dry weight in decellularized muscle), LatB decellularization is increasingly preferred in studies that are highly dependent on preserving dECM bioactivity. However, unlike SDS, it has been suggested that the LatB protocol does not scale well and is less effective at decellularizing larger tissues (Naik et al., 2020). Naik et al. demonstrated that the overall difference in tissue size alters the effectiveness of the decellularization reagents (Naik et al., 2020) and hence, a milder LatB protocol is insufficient in completely eliminating nuclear materials in larger whole human muscles as compared to smaller whole murine muscles.

**FIGURE 2:**
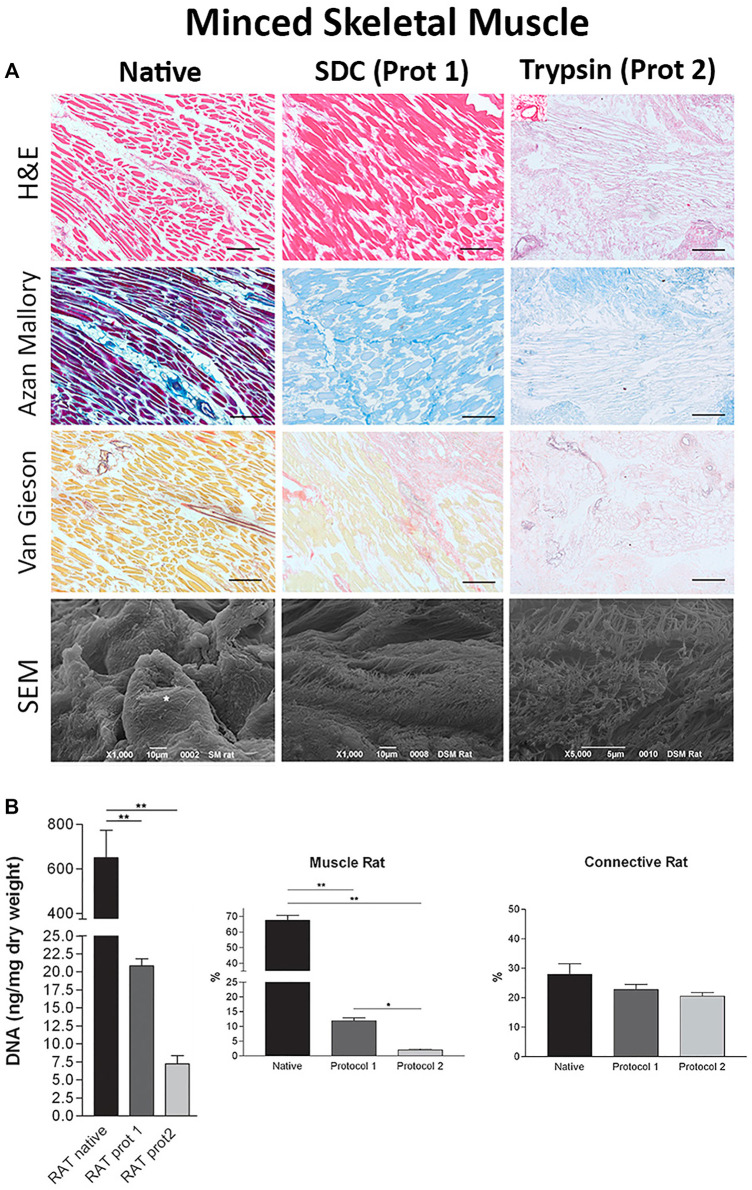
Minced tissue decellularization protocols and outcomes **(A)** Tissue morphology from top to bottom: H&E staining, Azan Mallory stains, Van Gieson stains, and SEM images of minced tissues before (Native, column 1) and after decellularization with SDC (Protocol 1, SDC + DNase I, column 2) and Trypsin (Protocol 2, Trypsin/EDTA + Triton X-NH4OH, column 3) protocols **(B)** DNA, muscle content, and connective tissue content before and after decellularization using protocol one and protocol 2. Figures A,B are adapted from “Decellularized Human Skeletal Muscle as Biologic scaffold for Reconstructive Surgery” by Porzionato et al. (2015) licensed under CC BY 4.0.

The decellularization of skeletal muscle is generally approached using either minced or whole tissues, each with their own corresponding advantages and disadvantages. Smaller minced tissues provide a larger surface area for cell lysis compared to larger whole tissues which aides in the effective removal of nuclear material but may sacrifice the spatial context of their original anatomical structures (Figure 2A,B). Conversely, decellularized whole tissues preserve the greater structure of the native tissue, but due to diffusion limitations, whole tissue protocols often require a harsher treatment agent, or the use of perfusion techniques to avoid extended decellularization times. However, the end application of the decellularized tissue can help determine which format is most appropriate. Whole tissue decellularization is useful in whole organ transplantation or in the replacement of large sections of injured skeletal muscle, whereas minced tissue decellularization is often used when the macrostructure is not a priority such as in bioink production for 3D printing. Because it is less vital to preserve the ECM spatial architecture for bioink production, these protocols often utilize higher concentrations of SDS (≥1% SDS), compared to whole skeletal muscle decellularization, which is usually performed with perfused 0.25% SDS or LatB (Choi et al., 2016; Kim et al., 2020; Lee et al., 2020; Jin et al., 2021a; Jin et al., 2021b).

In addition to the main decellularizing reagents, many protocols include supplementary chemicals to ensure complete decellularization. For instance, DNase is frequently used after the primary decellularization step to further assist in the removal of residual DNA (Gillies et al., 2011; Zhang et al., 2016; Urciuolo et al., 2018). Likewise, chloroform and isopropyl alcohol are regularly used to extract unwanted lipids from the native tissue before decellularization (Wolf et al., 2012; Ungerleider et al., 2015). In the final stages of the protocol, once a tissue has been decellularized, washes with sterilizing and anti-mycotic agents such as penicillin and streptomycin, (Merritt et al., 2010; Kasukonis B. et al., 2016; Kasukonis B. M. et al., 2016; Jin et al., 2021a; Jin et al., 2021b), peracetic acid, (Wolf et al., 2012; Zhang et al., 2016; Choi et al., 2019), and ethanol (Choi et al., 2019) are used. Lastly, many groups administer EDTA in series or in combination with the first few decellularizing agents to help prevent clot formation as the blood is removed from the tissue vasculature (Gillies et al., 2011; Miranda et al., 2021). Although these supplementary chemicals contribute to a more complete decellularization, they may also impact the ECM retention, myofiber morphology, and overall structural integrity of decellularized tissues. Therefore, the rule of thumb is to use the fewest agents possible and the shortest decellularization times to achieve the targeted dECM properties.

## 3 Biophysical Properties: ECM Composition

**FIGURE 3:**
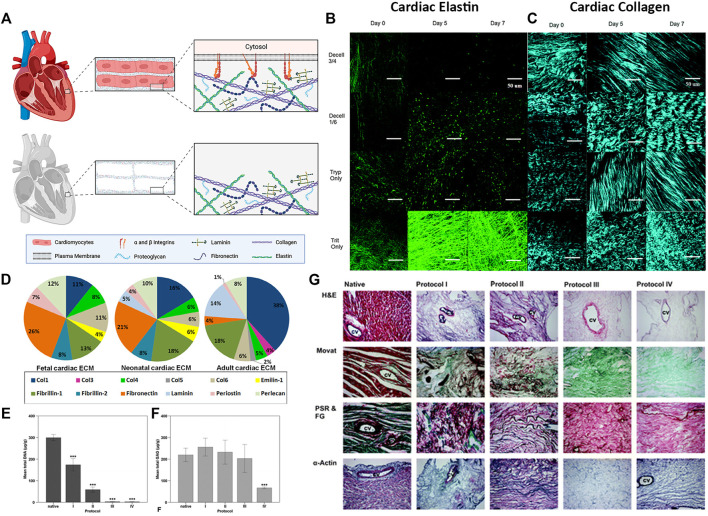
Cardiac extracellular matrix composition **(A)** Schematic of cardiac tissue before and after decellularization **(B–C)** Extracellular matrix microstructure of decellularized whole porcine hearts following four different decellularization protocols. Decell 3/4: Trypsin for 3 days followed by Triton for 4 days. Decell 1/6: Trypsin 1 day, Triton 6 days. Tryp Only: Trypsin 7 days. Trit Only: Triton 7 days. Scale bar 50 µm **(B)** Elastin microstructure. Two photon fluorescence **(C)** Collagen microstructure. Second harmonic generation imaging **(D–F)** Rat myocardial ECM morphology and composition before (native) and after whole heart decellularization using four different decellularization protocols. Briefly, Protocol I: SDS and Triton. Protocol II: Trypsin, EDTA, deoxycholic acid (DCA), and acetic acid. Protocol III: Glycerol, NaN_3_, EDTA, NaCl, DCA, SDS, and Triton. Protocol IV: SDS, DCA, NaN_3_, Glycerol, EDTA, Saponin, DNase I, and MgCl **(D)** The 15 most abundant proteins in cardiac ECM measured by liquid chromatography–mass spectrometry (LC-MS/MS) at each developmental age **(E)** Mean DNA content and **(F)** mean GAG content of the native whole rat heart before (native) and after decellularization using protocols I - IV **(G)** Rat myocardial ECM morphology from top to bottom: Hematoxylin and Eosin (H&E) staining, Movat Pentachrome (Movat), picrosirius red and fast green (PSR & FG), and alpha-actin of rat hearts before (Native, column 1) and after whole heart decellularization (Protocols I–IV, columns 2—5). CV, coronary vessel. ×100 magnification. Figure A is created using BioRender.com. Figures B, C are adapted with permission from Merna et al. (2013). Figure D is adapted with permission from Williams et al. (2014). Figures E–G are adapted with permission from Akhyari et al. (2011).

The ECM is composed of a highly complex network of macromolecules that provide a supportive structural and functional niche for the surrounding cells. While the composition and respective biological functions of ECM are still being investigated, the components of ECM can be broken down into three main groups: glycoproteins, GAGs, and proteoglycans (Figure 3A). Glycoproteins, such as collagen, elastin, laminin, and fibronectin, make up the bulk of the ECM and provide the primary structural support and binding sites for cells (Silva et al., 2016) (Figure 3B,C). GAGs play a crucial role in modulating biochemical processes such as cell proliferation, migration, and wound repair (Puri et al., 2020). Proteoglycans have GAGs bound to their core and are one of the most complex and multifunctional molecules in cell biology (Walimbe and Panitch, 2020). Some of the reported functions of proteoglycans include growth factor sequestering, control of collagen fibrillogenesis, regulation of angiogenesis, and modulation of cell adhesion, proliferation, and pathway regulation (Walimbe and Panitch, 2020). The ECM is a highly dynamic structure that is constantly changing in response to environmental cues from growth, development, disease, injury, and repair.

### 3.1 Cardiac dECM Composition

The investigation of cardiac ECM composition was historically challenging due to the low solubility of the tissue’s macromolecules and their post-translational modifications (Chang et al., 2016). However, advancements in ECM protein enrichment and technology have enabled a more complete investigation of cardiac ECM (Lindsey et al., 2018). Johnson et al. identified over 200 distinct cardiac ECM proteins in decellularized human hearts, the most prominent being collagen I, collagen IV, fibrillin 1, collagen V, collagen VI, laminin, and perlecan (Johnson et al., 2016).

The composition of cardiac ECM is also dependent on age. Multiple groups have compared the composition of cardiac ECM at varying stages of development and maturation (Williams et al., 2014; Fong et al., 2016; Johnson et al., 2016; Silva et al., 2016). Of the 15 most abundant cardiac proteins detected in rat hearts, adult ECM contained 38% collagen I, 18% fibrillin, 14% laminin, 8% perlecan, while fetal hearts contained 26% fibronectin, 13% fibrillin, 12% perlecan, 11% collagen I (Figure 3D) (Williams et al., 2014). Furthermore, fibrillin-2 and emilin-1 were among the top 15 most abundant proteins in fetal cardiac ECM, but not in adult cardiac ECM. These differences in composition provide insight into the supportive niche of the developing heart and may serve as a blueprint for ideal ECM mixtures to use for modulating regeneration.

#### 3.1.1. How Different Protocols Affect Cardiac dECM Composition

Maintaining cardiac ECM proteins while thoroughly removing native DNA and cellular fragments is crucial for the success of many downstream applications. Most groups validate their decellularization protocol by quantifying the total DNA and GAG content that is left following decellularization. It is widely accepted that a protocol that results in less than 50 ng of double-stranded DNA per mg of tissue dry weight is effective in clearing the tissue of nuclear debris, although it should be noted that many groups measure and report residual DNA content differently (concentration, fragment length, staining) (Gilpin and Yang, 2017). However, reagents that thoroughly remove DNA and cellular debris often also disrupt GAG content. Akhyari et al. used a long duration protocol (5.7 days) with 1% SDS +1% DCA that produced tissues with less than 0.5% residual DNA, but significantly decreased GAGs by almost 70% compared to native content (Figure 3E–G) (Akhyari et al., 2011). In contrast, when the same group used a short duration protocol (4.75 h) with 1% SDS and 1% Triton X-100, the resulting tissues showed no decrease in GAGs (255.53 μg/g vs 219.33 μg/g native); however, approximately 13.9% residual DNA was still present.

Fortunately, there are combination protocols that have been shown to strike a balance between GAG retention and DNA removal. Wainwright et al. used a mixture of chemical, physical, and enzymatic decellularization techniques to maintain GAGs while also adequately removing residual DNA (0.66 ng DNA/mg vs 8.48 ng DNA/mg native, a 92% decrease) (Wainwright et al., 2010). A multi-step protocol by Lu et al. used trypsin, triton, cycles of freeze/thaw, SDS, deoxycholic acid, peracetic acid, and ethanol to produce ECM with preserved 3D architecture and filament structure while still achieving ∼97% DNA removal (Lu et al., 2013). Alternatively, an adequate retention/removal balance has been achieved with simpler protocols that use classically damaging reagents for a short amount of time, followed by thorough washing to clear the matrix. This includes one of the most published cardiac decellularization approaches which uses 1% SDS and 1% Triton X-100 with several deionized water or PBS rinses (Ott et al., 2008; Robertson et al., 2014; Williams et al., 2014; Hochman-Mendez et al., 2020; Wang et al., 2020; Basara et al., 2021; Mousavi et al., 2021; Tsui et al., 2021). This approach has been demonstrated to have an adequate balance of DNA removal while maintaining ECM composition.

#### 3.1.2 How Cardiac dECM Composition Affects Cell Behavior In Vitro

The ECM is a potent modulator of cell behavior and recent efforts in the field of cardiac dECM have focused on the use of this material to guide cells *in vitro*. Through the sophisticated regulation of soluble factors and cell signaling pathways, the ECM components can guide proliferation, fate specification, and migration. (Mesquita et al., 2021). However, there has been a gradual shift away from classical whole organ decellularization towards protocols that utilize ground or solubilized cardiac ECM. This pivot was thought to have been fueled by growing evidence that the concentration and bioactivity of dECM components played a greater role in guiding recellularization than the overall ultrastructure.

A well-known bottleneck of cardiac tissue engineering has been the limited proliferation potential of cardiomyocytes. However, ground or solubilized cardiac ECM serves as an instructive cell culture additive that can enhance cardiomyocyte proliferation. Williams et al. cultured ventricular cells on solubilized dECM and analyzed attachment, proliferation, and maturation (Williams et al., 2014). They found that fetal cardiac ECM was associated with greater cardiomyocyte adherence and proliferation in comparison to the Poly-l-Lysine (PLL) control and adult ECM. ECM components such as periostin and collagen IV were more abundant in the fetal ECM compared to adult, suggesting that these likely played a decisive role in enhancing myocyte proliferation.

Cardiac ECM has also been shown to improve cardiomyocyte differentiation and maturation (Das et al., 2019; Hochman-Mendez et al., 2020; Mesquita et al., 2021). Hochman-Mendez et al. cultured human embryonic stem cell-derived cardiomyocytes (hESC-CM) with dECM particles (Hochman-Mendez et al., 2020). hESC-CMs attached to the basement membrane and demonstrated enhanced maturation characteristics such electrical coupling, gap junctions, and increased sarcomere lengths. Das et al. showed that neonatal rat cardiomyocytes grown in dECM bioink rather than a single ECM protein of just collagen alone, facilitated a cell-material cross-talk that drove cell morphology and function (Das et al., 2019).

Not only can cardiac ECM improve maturation of stem cell-derived cardiomyocytes, but it can also guide phenotype specificity. Most cardiomyocyte differentiation protocols generate ventricular-like cells, but atrial-like cardiomyocytes are also needed for therapeutics and disease modeling. In order to generate atrial-like cardiomyocytes, high concentrations of retinoic acid are used; however, this agent is unstable in serum-free media, making standardized differentiation protocols difficult. Mesquita et al. demonstrated the use of chamber-specific powdered ECM to drive cardiomyocyte subtype differentiation (Mesquita et al., 2021). When human iPSCs were cultured with human atrial dECM particles, the cells expressed more atrial-like characteristics as verified by proteomics, action potentials, and gene expression. Similarly, human iPSCs cultured with ventricular ECM particles resulted in more mature ventricular CMs, as supported by the electrophysiological characterization, compared to iPSCs differentiated without ECM (Mesquita et al., 2021).

#### 3.1.3 How Cardiac dECM Composition Affects Cell Regeneration In Vivo

Historically, cardiac dECM was viewed as a potential therapeutic in the form of a transplantable whole organ. The idea was to recellularize a full dECM heart with patient-specific cells to provide an autologous organ for transplantation, eliminating the need for lifelong anti-rejection medication. The cell requirements needed to repopulate an entire human heart is a major rate-limiting step and led to the use of dECM segments for use as cardiac patches, which showed promise as a post myocardial infarction (MI) treatment. Shah et al. implanted a 300 μm slice of decellularized porcine myocardium with or without rat adipose stem cells into rats 20 min after coronary artery ligation (Shah et al., 2018). Hearts treated with the dECM patch had increased vascularization and significantly more cells present compared to cells delivered via direct injection after 1 week. While the use of intact dECM is still explored for patches, other forms of dECM are being developed for greater control over the decellularization and recellularization processes.

It is well-understood that the retention of ECM protein composition and integrity are essential for cell adherence, proliferation, and maturation. However, preserving protein bioactivity may come at the expense of residual DNA and cell fragments, which can elicit an immune response, thereby limiting the ECMs regenerative potential (Keane et al., 2012). Fortunately, *in vivo* rat and mouse studies primarily use cardiac dECM in a ground or solubilized form, which enables more efficient removal of cell debris due to optimizing surface area and diffusion distances (Singelyn et al., 2012; Wang et al., 2019; Wang et al., 2020). Without the need to keep the whole heart architecture intact, protocols using minced, ground, or solubilized ECM can more thoroughly remove residual DNA and cell fragments without inducing significant changes to the overall ECM composition.

dECM injectables have demonstrated translational efficacy in small animal models of cardiac injury (Singelyn et al., 2012). Wang, Z. et al. injected adult or neonatal mouse dECM suspended in saline into the epicardium of mice immediately following induced MI (Wang et al., 2019). Neonatal dECM was shown to improve cardiac function, reduce fibrosis, and promote angiogenesis. Adult dECM did not share the regenerative capabilities of the neonatal dECM, and in many instances, performed no better than the saline control. This study, and similar studies that use injectable dECMs, highlight the functional benefits of solubilized dECM as a material to mitigate the negative remodeling that often follows MI. Moreover, this underscores a paradigm shift away from conventional adult-derived ECM materials and towards a future that looks further into the advantages and understanding of neonatal-derived dECMs.

Another form of solubilized dECM that has been growing in popularity is bioinks that are used to generate cardiac patches. Bejleri et al. bioprinted a GelMA-dECM patch and implanted it onto healthy rat epicardium for 14 days (Bejleri et al., 2018). Histological analysis showed that the patch became vascularized and integrated with the host myocardium. Jang et al. also bioprinted a cardiac patch containing human dECM and polycaprolactone (PCL), with alternating layers of cardiac progenitor cells and mesenchymal stem cells (Jang et al., 2017). The patches were first implanted subcutaneously into the abdomen of the rat for host vascularization. After 28 days, the patch was explanted and then implanted onto the epicardium of rats that had an induced MI 7 days prior. This alternating layered approach led to improved cardiac function, attenuated fibrosis, greater cell migration out from the transplanted patch into the damaged tissue, and enhanced vasculogenesis and myogenesis. These works demonstrate how cardiac dECM can be solubilized and used as a supportive niche to promote a pro-regenerative and mitigate a pro-fibrotic healing response following cardiac injury.

Lastly, the cardiac dECM is rich with cytokines and small molecules that modulate angiogenesis, survival, cell recruitment and the cellular response to inflammation. Decellularized porcine myocardium was found to contain vascular growth factors such as VEGF-A, VEGF-C, and FGF-1, cardiokines including PDGF, HGF, and endoglin, as well as factors involved in the inflammation such as G-CSF and IL-8 (Methe et al., 2014). While these residual factors modulate a range of pro-survival and pro-recruitment functions, they also exhibit batch-to-batch variability and present an ongoing challenge in the standardization of decellularization protocols.

### 3.2 Skeletal Muscle dECM Composition

The ECM of native skeletal muscle is composed of a combination of proteins and proteoglycans such as collagen, elastin, laminin, fibronectin and GAGs (Csapo et al., 2020). These components serve as modulatory binding sites, structural materials, and gene regulators for the native muscle and if preserved correctly, can serve these same functions as a decellularized material for skeletal muscle regenerative applications (Csapo et al., 2020). However, due to the wide range of decellularization protocols, the resulting dECM composition varies and hence the regenerative capacity of these materials will also vary.

#### 3.2.1 How Different Protocols Affect Skeletal Muscle dECM Composition and Cell Interactions - Trypsin

Trypsin is a serine protease secreted by the pancreas to aid in the digestion of proteins (Miersch et al., 2018). In cell culture, trypsin is commonly used for detaching adherent cells during cell passaging (Sharma et al., 2019) as it disrupts the cell-ECM bonds. For this reason, trypsin is also used as a dissociation agent in skeletal muscle decellularization. However, due to its strong digestive properties, trypsin is also effective in removing essential ECM components like collagen and GAGs which present a challenge for downstream applications that rely on interactions with these components.

Wolf et al. established a decellularization protocol for whole muscles using 0.1% Trypsin, 2% SDC and 1% Triton X-100 (Wolf et al., 2012). ECM characterization using Herovici’s and Movat’s Pentachrome stains detected the presence of collagen and elastin, while immunostains of the basement membrane showed the preservation of laminin, type IV collagen, and fibronectin. Although the stains showed some ECM presence, the stains also revealed the dysmorphism of the physical structures in decellularized skeletal muscles. Furthermore, only 20% of sulfated GAGs (sGAGs) were retained in decellularized skeletal muscles when compared to native skeletal muscles. When these muscle dECMs were seeded with human perivascular stem cells, NIH 3T3 mouse fibroblasts, and C2C12 mouse myoblasts, cellular proliferation was observed on the surface of the decellularized tissue for all cell types. Additionally, the cultured C2C12s further differentiated into mature myotubes. In contrast, cellular metabolism quantified using Alamar Blue assay indicated higher metabolic activity in the tissue culture flask control compared to cells grown on the decellularized scaffold. *In vivo* implantation of the decellularized scaffold in a rat abdominal wall defect showed dense accumulation of regenerating cells, angiogenesis, and myogenesis around the scaffold surface as shown by histological analyses. Corona et al. used a similar combination of reagents on whole rat tibialis anterior (TA) muscles and showed enhanced cell proliferation of muscle satellite cells *in vitro*, but limited myogenesis when implanted into a volumetric muscle loss (VML) injury (Corona et al., 2013).

In contrast, greater *in vivo* healing outcomes were reported by Zhang et al. who combined 0.02% trypsin with 0.1% SDS and 1% Triton X-100 to perfusion decellularize whole porcine skeletal muscles (Zhang et al., 2016). ECM characterization of the decellularized tissue validated the retention of laminin, type IV collagen, fibronectin, and elastin. Similarly, sGAG quantification of native versus decellularized tissues corresponded to a preservation of roughly 20% sGAGs in the decellularized tissues. The culture and differentiation of C2C12 myoblasts on the dECM scaffold demonstrated improved myogenesis including greater fiber diameter, fusion index, and increased metabolism compared to the tissue culture plastic controls. In a VML injury model involving a rat abdominal wall defect, the transplanted dECM scaffold showed abundant cellular infiltration around the surface of the implant as well as moderate neo-muscle fiber presence up to 5 mm away from the defect. Neovascularization of the skeletal muscle dECM was also greater compared to the controls. Taken together, there is both utility and limitations of using a trypsin-based decellularization agent in which survival and proliferation of cells is supported *in vitro* but structural dysmorphism and loss of GAGs may impact *in vivo* applications.

#### 3.2.2 How Different Protocols Affect Skeletal Muscle dECM Composition and Cell Interaction - SDS

A well strategized SDS treatment can be extremely effective at removing cellular debris, while still retaining ECM components. In an effort to restore normal muscle function following a severe muscle injury, Lee et al. decellularized minced rabbit skeletal muscles using 1% SDS to create a dECM scaffold (Lee et al., 2020). A compositional analysis of the resulting ECM in the decellularized tissue showed the retention of approximately 50% collagen, 74% elastin, and 83% GAGs when compared to native skeletal muscle. When seeded with C2C12 mouse myoblasts, the cellularized constructs maintained a 90% cell viability throughout the 7 days of culture. Alamar Blue assessment of metabolic activity of the cells in the muscle dECM demonstrated an increase in cell proliferation when compared to collagen controls. Additionally, greater *in vitro* myogenesis (myotube formation and maturation) was observed on the dECM scaffolds compared to controls. In a rabbit model of TA VML injury, treatment with the dECM scaffold was associated with a greater number of regenerated muscle fibers when compared to the collagen implant. Therefore, despite the reputation of SDS as a disruptive agent, dECM produced from an SDS protocol has been used for successful *in situ* muscle regeneration.

The decellularization of whole muscle tissues presents additional challenges due to the large volume of tissue that must interface with a decellularization agent to effectively remove cellular components. Protocols that aim to decellularize whole muscles typically include some form of SDS. To decellularize whole rat muscles, Kasukonis et al. employed a 1% SDS perfusion decellularization protocol (Kasukonis B. M. et al., 2016). ECM characterization of the decellularized tissue showed an approximately 90% retention of both collagen and GAGs. *In vitro*, the dECM supported C2C12 proliferation and *in vivo* implantation of the dECM scaffold with and without the addition of minced muscle autografts in a rat VML injury model resulted in reduced fibrosis when compared to the VML only control (Kasukonis B. et al., 2016; Kasukonis B. M. et al., 2016).

**FIGURE 4:**
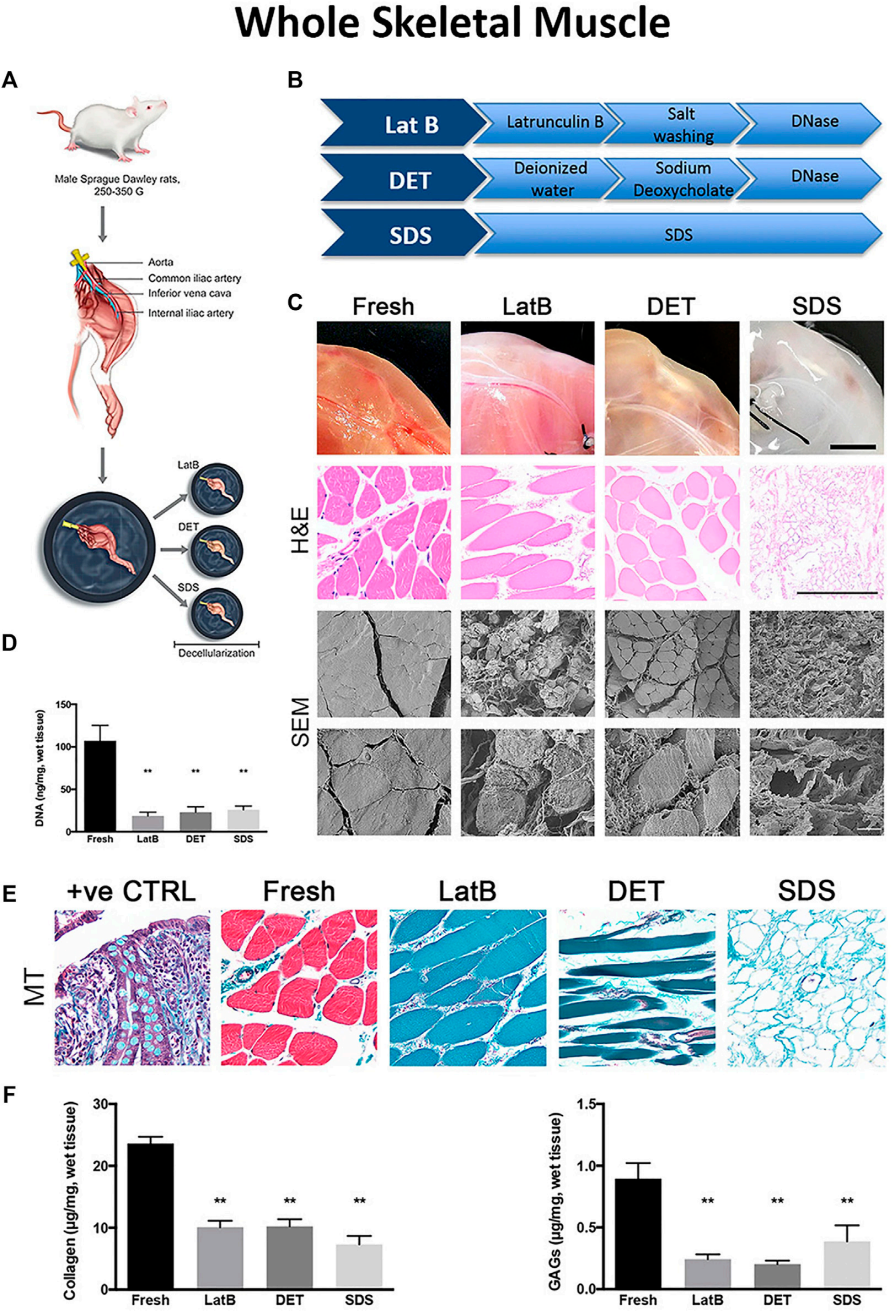
Differences in decellularization protocols determine dECM properties in whole tissues **(A)** Schematic of tissue harvest from a rat hindlimb for whole muscle decellularization **(B)** A breakdown of three example decellularization protocols used in whole tissue decellularization **(C)** Final DNA content after whole tissue decellularization using three different decellularization protocols **(D)** Tissue morphology from top to bottom: Macroscopic tissue images, Hematoxylin and Eosin (H&E) staining, and Scanning Election Microscope (SEM) images of whole tissues before (Fresh, first column) and after decellularization with LatB (second column), DET (third column), and SDS (fourth column) protocols **(E)** Massons Trichrome (MT) staining of whole tissue after decellularization **(F)** ECM content of collagen and GAGs remaining in decellularized whole tissues compared to fresh tissues. Figures A–F are adapted from “Decellularised skeletal muscles allow functional muscle regeneration by promoting host cell migration” by Urciuolo et al. (2018) licensed under CC BY 4.0.

To characterize the effects of three different decellularization protocols, Urciuolo et al. employed a perfusion treatment for rat hind limbs using one of three agents: 1) LatB; 2) 4% SDC; or 3) 0.25% SDS (Urciuolo et al., 2018) (Figure 4A,B). Compared to native muscles, both H&E and SEM analyses displayed different histological characteristics as a function of decellularization agent (Figure 4C). Although the residual DNA content was statically lower than native tissues for all three treatments (Figure 4D), marked differences in global tissue morphology and myofiber structure was evident. Tissue architecture was extensively preserved in both the LatB and SDC treatment, whereas myofiber integrity was widely compromised in the SDS treatment (Figure 4E). Both the LatB and SDC protocols preserved about 50% of the native collagen content while the SDS protocol retained slightly less collagen at about 40% (Figure 4F). However, the same SDS treatment yielded tissues with a better retention of GAGs (∼45%), compared to approximately a 25% retention for both the LatB and SDC treatments (Figure 4F). Angiogenic cytokine content was also quantified in the three dECMs and revealed a 30 and 25% retention of VEGF using the SDS and SDC protocols respectively, while the LatB protocol showed almost no residual VEGF when compared to native skeletal muscles. Additionally, SDS preserved the greatest amount of IGF-1 (60% retainment) while both LatB and SDC correlated with about 33% compared to native tissues. To further test the regenerative potential of the decellularized tissues, each scaffold was transplanted into a murine extensor digitorum longus (EDL) VML injury model. All sites of implantation showed signs of tissue regeneration with partially restored muscle strength. Despite some differences in dECM composition, *in vivo* application of this material resulted in similar regenerative outcomes.

#### 3.2.3 How Different Protocols Affect Skeletal Muscle dECM Composition and Cell Interaction - LatB

A non-proteolytic, non-detergent based, decellularization method was developed by Gillies et al. for whole skeletal muscle decellularization (Gillies et al., 2011). The protocol consisted of LatB, potassium chloride, potassium iodide, and DNase, applied to whole mice TA muscles. LatB functions by depolymerizing the actin and myosin filaments that are the structural supports of skeletal muscle, making cells more vulnerable to the effects of potassium chloride and potassium iodide salts which are hyper- and hypotonic solutions. Most notably, major ECM components were preserved. Amongst the ECM components, 60% of GAGs and 100% of collagen remained in the decellularized tissue. Moreover, when seeded with C2C12 mouse myoblasts, the skeletal muscle dECM supported cell survival both on the surface and inside the scaffold.

Using this same LatB protocol, Fishman et al. decellularized the cricoarytenoid dorsalis muscles harvested from the larynx of adult rabbits (Fishman et al., 2012). The non-proteolytic, non-detergent protocol actively preserved several major ECM components, which corresponded with the retainment of about 75% collagen, 65% GAGs, preservation of elastin, and low presence of MHC classes I and II antigens. To test the biocompatibility and regenerative properties of the acellular tissue, the skeletal muscle dECM was transplanted into immunocompetent rats with a TA injury (Fishman et al., 2013). Compared to the control PCL scaffolds, the dECM scaffolds produced better cell survival and host integration.

Overall, the LatB decellularization approach pioneered by Gillies et al. is emerging as a viable alternative to the traditional SDS and trypsin treatments. Although a more mild protocol, LatB effectively preserves ECM components and may be an efficient approach for the decellularization of whole skeletal muscle decellularization without the need for perfusion bioreactors.

## 4 Biophysical Properties: Topography

The ECM is an elegant example of form supporting function. The ultrastructure of an ECM niche is dictated by the needs of the cells that it is designed to support. In the case of cardiac and skeletal muscle, this means that the tissue ECM is highly organized and anisotropic to enable fast and uniform transfer of contractile forces across the entire tissue. The topographical features of the ECM are present from the macrostructure down through the sub-cellular organization of fibrillar proteins. The spatial patterning of these structures provides a supportive niche that can direct cell organization, behavior, and function. Topographical patterning using dECM materials is generally performed using two different approaches: 1) use of the intact native matrix with original architecture; 2) use of solubilized matrix that is then printed into specific patterns to guide cell behavior.

### 4.1 Cardiac dECM Topography

The theme of alignment is pervasive throughout all levels of the cardiac tissue structure from the parallel orientation of the myocardial macrostructure to the sub-micron bands of the sarcomeres (Carson et al., 2016). Much of the structural alignment that is seen in cardiac tissue is primarily dictated by fibronectin and collagen fibers (Batalov et al., 2021), which have diameters ranging from 30 to 120 nm (Carson et al., 2016). Within these fibers are the integrin-binding domains that interact with cells to facilitate attachment and thereby are responsible for guiding cellular alignment. In fibronectin, the most common domain is the arginine-glycine-aspartate peptide (RGD) that facilitates binding through the α5β1 integrin complex (Bellis, 2011). Collagen also has RGD sequences, but these sites are largely inaccessible due to the conformation unless the collagen is cleaved by MMPs or denatured (Bellis, 2011). Other important binding players located on cell membranes are the α1β1 integrin complex, which primarily binds to collagen type IV, and the α2β2 integrin complex, which predominantly binds to collagen type I (Boraschi-Diaz et al., 2017). The accessibility of the integrin-binding domains following decellularization plays a decisive role in guiding cardiac cell binding and cytoskeletal organization. Therefore, the decellularization protocols aimed at generating dECM with specific topographical features, must prioritize the preservation of the ECM components that house these cell-binding domains.

#### 4.1.1 How Different Decellularization Protocols Affect Cardiac dECM Topography

To enable the study of dECM topography, decellularization protocols must avoid disruption of key ECM proteins such as fibronectin and collagen fibers, which contain essential integrin-mediated binding domains. Protocols that remove cells via cell swelling and rupture have been shown to physically disrupt the spatial environment, and repeated freeze-thaw cycles can be severely damaging to fibrillar proteins, leading to inconsistent fiber sizes (Behmer Hansen et al., 2021). Similarly, extended exposure to reagents such as trypsin, SDS, and Triton X-100 can disrupt glycoprotein microstructure which is important for cell adhesion and growth factor regulation (Wilgus, 2012; Gilpin and Yang, 2017). For example, Merna et al. found that hearts decellularized with a combination of trypsin and triton thoroughly removed DNA (<1 ng/mg, ∼90% removal), but significantly disrupted the elastin architecture (Figure 3B) (Merna et al., 2013). In contrast, a triton-only regimen had less of an effect on elastin content and maintained crimped collagen fibers; however, the protocol resulted in only about 40% decellularization based on residual DNA content.

Fortunately, the most common cardiac decellularization approach of 1% SDS and 1% Triton X-100 is effective in the removal of cells and cellular debris without significant disruption to the ECM. (Ott et al., 2008; Robertson et al., 2014; Williams et al., 2014; Hochman-Mendez et al., 2020; Wang et al., 2020; Basara et al., 2021; Mousavi et al., 2021; Tsui et al., 2021). Using this classic decellularization approach, Hochman-Mendez et al. even confirmed that the wave-like topography of laminin in the cardiomyocyte basement membrane was preserved (Hochman-Mendez et al., 2020). However, the precise level of ECM feature preservation required for adequate cell binding, growth, and patterning still remains to be extensively characterized.

#### 4.1.2 How Cardiac dECM Topography Affects Cell Behavior In Vitro

A significant body of work has been done to study native topography and to characterize the drivers of cardiomyocyte alignment. Micropatterning, nanofiber electrospun scaffolds, flexible posts, and electrical fields are just some of the methods used to guide alignment *in vitro* (Au et al., 2007; Carson et al., 2016; Leonard et al., 2018; Suh et al., 2020; Batalov et al., 2021). While it is well understood that the orientation and elongation of cardiac cells is essential for maturation and proper contractile function, the field has yet to fully elucidate the underlying biophysical, mechanical, and molecular mechanisms that facilitate alignment.

To better understand these complex relationships, Schwan et al. developed an engineered heart tissue (EHT) system from laser cut, decellularized porcine myocardial sheets (Schwan et al., 2016). A major insight from this work was that local matrix cues from the EHT were more influential in guiding cellular alignment than macroscopic cues such as applied load or stiffness, which are historically potent modulators of alignment in isotropic gels (Schwan et al., 2016). When the EHT was recellularized with neonatal rat ventricular myocytes, human embryonic stem cell derived cardiomyocytes, or human induced pluripotent stem cell derived cardiomyocytes (iPSC-CMs), the resulting construct displayed spontaneous beating, cellular alignment, organized sarcomeres, and gap junctions. They found that the mechanical properties, such as peak twitch stress, were dictated by the orientation of the fibers and could be used to recapitulate the contractile anisotropy displayed by native tissues. Moreover, when they compared the EHT to another anisotropic scaffold, electrospun gelatin, they found that the EHT had a stronger peak stress, suggesting that the dECM possess a diverse multitude of biomechanical factors that similarly patterned synthetic materials cannot recapitulate.

#### 4.1.3 How Cardiac dECM Topography Affects Cell Regeneration In Vivo

The vast majority of *in vivo* studies that use cardiac dECM, do so by using the material in a solubilized form. While topography and patterning of dECM *in vitro* may have provided evidence of guiding cellular and biomechanical outcomes, the story *in vivo* is different. Cardiac dECM injectable therapeutics have had promising results (Singelyn et al., 2012; Wang et al., 2019; Wang et al., 2020). However, there are a few studies that showed the benefit of using dECM with preserved topography as a tool for enhancing cell recruitment or delivery in small animal injury models. Sarig et al. implanted decellularized porcine cardiac patches into rats following induced MI and observed recruitment of native macrophages and cardiomyocyte progenitor cells (CPCs) (Sarig et al., 2016). Recruited CPCs organized into fiber-like clusters with partial striation and positive staining for gap junction marker Connexin43. Sarig et al. concluded that their dECM patch, without recellularization, improved contractility, ejection fraction, and cardiac remodeling following MI (Sarig et al., 2016).

Like Sarig et al., Wang, Q. et al. used intact rat decellularized ECM patches for treatment post MI in a rat model, but also directly compared acellular dECM patches to dECM patches cultured with human iPSC-CMs and CD90^+^ nonmyocytes (Wang et al., 2016). They found that recellularized patches significantly improved ejection fraction and vascular density, while decreasing infarct size in comparison to acellular dECM patches. However, their recellularization did not produce adequate cellular alignment or cardiomyocyte maturation and they did not study recruitment of host cells. Taken together, *in vivo* application of cardiac dECM is largely driven by modulation of the regenerative niche by the ECM components and, to a much lesser extent, by any specific surface patterning or topographical features.

### 4.2 Skeletal Muscle dECM Topography

**FIGURE 5:**
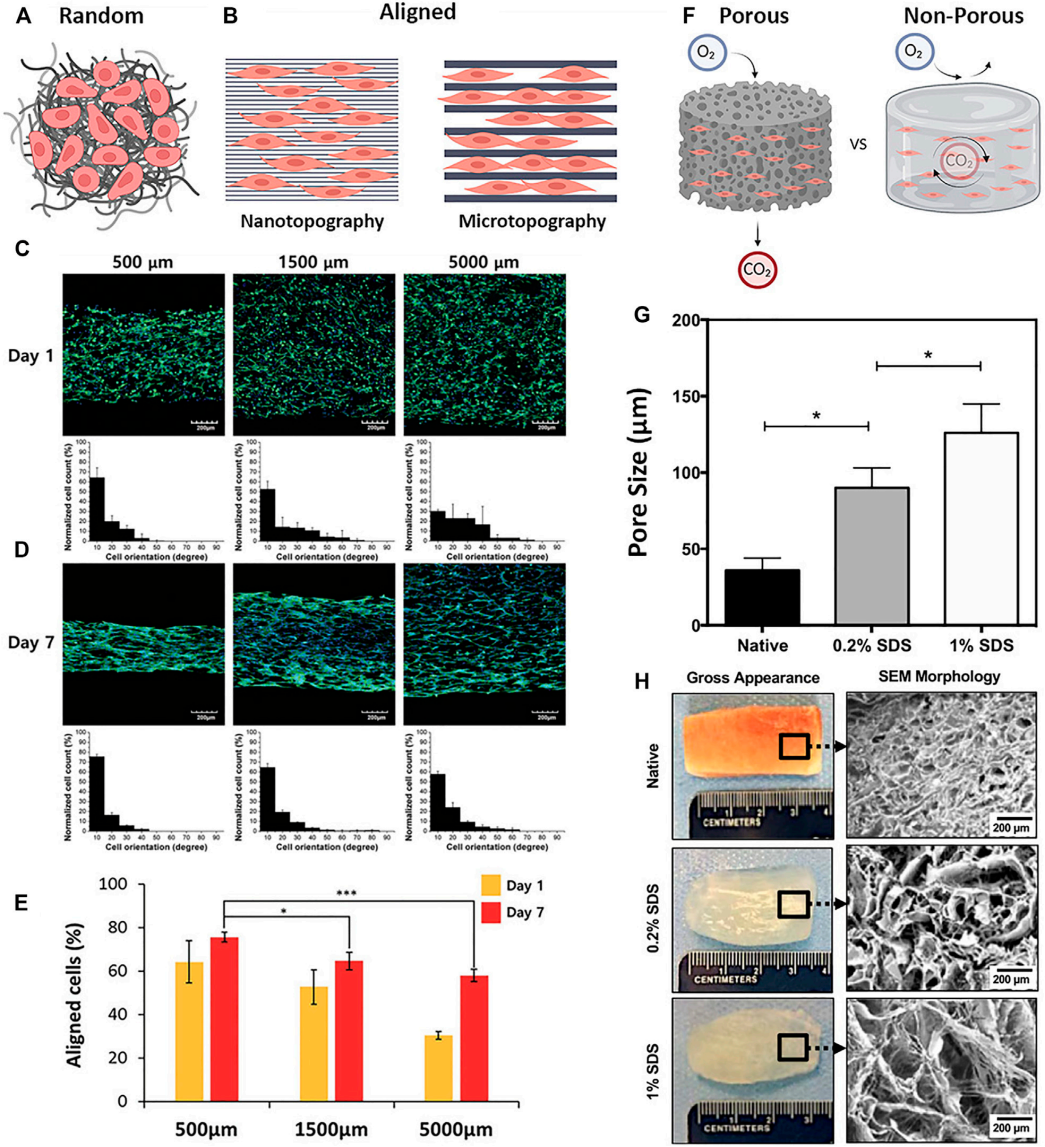
Patterning and topography in dECM biomaterials **(A)** Material topography with a random-orientation of fibers and cells **(B)** Nano- and micropatterned materials with parallel orientation of fibers or channels showing differences in cell guidance and interaction **(C–E)** C2C12 myoblasts cultured on 3D-printed line widths of 500 μm, 1,500 μm, and 5,000 µm on **(C)** day 1 and **(D)** day 7**;** cells were stained green for F-actin (Phalloidin), and blue for nuclei (DAPI) **(E)** Quantification of % aligned cells on the varying line widths at day 1 and 7 **(F)** Porous and Non-Porous properties of bulk materials differentially regulate the diffusion of gases and other soluble factors **(G)** Quantification of average pore sizes in native skeletal muscle tissue and decellularized scaffolds treated with 0.2% SDS and 1% SDS, respectively **(H)** Macroscopic and SEM images of native skeletal muscle and decellularized scaffolds treated with 0.2% SDS and 1% SDS, respectively. Figures A, B, F are created using BioRender.com. Figures C–E are adapted with permission from Choi et al. (2016). Figures G, H are adapted with permission from Shapiro et al. (2019).

Many studies that look at the topographical contribution of decellularized skeletal muscle aim to utilize and/or mimic the naturally aligned pattern of the ECM to guide myogenic cell differentiation. Similar to cardiac muscle, skeletal muscle is highly oriented along a common axis and is composed of functional myofiber units that are organized in parallel to maximize force contraction. Skeletal muscle is anisotropic and is made up of tightly bundled, aligned structures of densely-packed myofibers (Jana et al., 2016). In the field of skeletal muscle regeneration, differentiation of new myofibers along a uniform direction is a key feature of a successful biomimetic material and supportive niche. Furthermore, it has been widely demonstrated that topographical patterning of cells has a significant impact on morphology, behavior, and function (Nakayama et al., 2018; Nakayama et al., 2019). There are several reoccurring themes in the area of topographical patterning and skeletal muscle dECM that include the contribution of alignment/anisotropy, scale (micro versus nano), and porosity (Figure 5).

#### 4.2.1 How Skeletal Muscle dECM Topography Affects Cell Behavior- Alignment

The alignment-mediated cellular response is a key player in myogenesis and the development of functional myotubes and physiologically mimetic myofibers (Ostrovidov et al., 2014). Substrates with either aligned or randomly-patterned topography are often used to assess or enhance contact-driven myogenic fusion (Figure 5A). One approach that utilizes skeletal muscle dECM with controlled topographical patterning was demonstrated by Kim et al. using 3D bioprinting methods to produce a cell-laden, dECM-based structure with unique topographical cues (Kim et al., 2020). To test the effect of uniaxial topographical patterning, C2C12 mouse myoblasts were seeded onto either aligned or non-patterned dECM scaffolds and were cultured for 21 days. Significantly greater myoblast alignment, myotube formation, and myogenic gene expression (MyoD1, MYH2, Myog) was observed in the aligned patterned dECM scaffolds when compared with the non-patterned controls. In another alignment-focused study, through the use of elastomeric chips made from polydimethylsiloxane (PDMS), the kinetics of dECM fibrillogenesis was controlled to fabricate both aligned and non-patterned dECM hydrogel scaffolds loaded with induced skeletal muscle progenitor cells (iMPCs) (Jin et al., 2021a). iMPC-loaded aligned dECM hydrogel scaffolds showed increased cell alignment and expression of myogenic markers Mrf5, MyoD1, and Myog at day 10 of *in vitro* cell culture. *In vivo* implantation of both aligned and non-patterned iMPC-loaded 3D constructs in a VML quadriceps femoris mouse model demonstrated a significant reduction of fibrosis as well as greater *de novo* muscle regeneration in the aligned iMPC-loaded hydrogel scaffold compared to the non-patterned control. These combined studies highlight the ability of physical cell alignment to enhance myogenesis *in vitro* and muscle regeneration *in vivo*.

Hybrid dECM bioinks are another approach to guiding alignment-mediated muscle regeneration. Lee et al. created aligned and randomly-oriented bioprinted skeletal muscle constructs using photo-crosslinkable dECM bioink together with poly(vinyl alcohol) (PVA) to modulate topography. The self-aligned printed muscle constructs improved myogenic differentiation *in vitro* evidenced by the expression of mature myogenic markers (MYH4 and MYH2) and exhibited rapid restoration of muscle function *in vivo* in a rat tibialis anterior VML model (Lee et al., 2021). The aligned constructs showed increased myofiber formation, reduced fibrotic tissue area, significant MHC^+^ expression, and greater tetanic force production. Together, these studies underscore the modulatory role of dECM alignment in guiding myogenesis.

#### 4.2.2 How Skeletal Muscle dECM Topography Affects Cell Behavior- Scale

Topography can be modulated on the micro- or nano-scale to guide cell behavior and myogenesis; however, it is a highly contested topic as to which topographical scale is most effective at achieving the optimal cell response (Skoog et al., 2018) (Figure 5B). Because of the increasing accessibility of technologies such as 3D bioprinting, electrospinning, soft- and photolithography, patterning using lower resolution microscale topographies are more widely used compared to higher resolution nanoscale topographies (Chia and Wu, 2015). For example, Choi et al. fabricated micro-scale cell-laden dECM muscle constructs using 3D printing of dECM with line widths that ranged between 300 and 1,000 μm. The micropatterned 3D printed constructs demonstrated high cell viability and supported the formation of densely packed aligned-myotubes compared to non-patterned controls. Furthermore, to assess therapeutic efficacy, the 3D micropatterned muscle constructs were implanted into the defect site of a rat tibialis anterior VML injury and displayed *de novo* muscle regeneration and minimal fibrosis when compared to the non-patterned controls (Choi et al., 2019).

Studies have also been done using dECM with different pattern scales to determine the optimal fiber size for myogenesis *in vitro*. Using 3D bioprinting, Choi et al. constructed C2C12-laden scaffolds with fibers that ranged between 500 and 5,000 μm (Choi et al., 2016) (Figure 5C,D). It was found that the greatest cellular alignment, elongation, and mechanical strength was seen by cells cultured on scaffolds with the higher resolution range of 500 μm patterning (Figure 5E). Evidence that smaller topographies may lead to enhanced myogenesis was demonstrated by this same group using deep X-ray lithography to fabricate sinusoidal microscale patterns of dECM in the range of 20–80 μm (Choi et al., 2018). These wavy dECM surfaces induced myoblast alignment and enhanced myotube formation and differentiation.

Nano-scale ECM topographies have been shown to play a potent role in guiding cell morphology, phenotype, differentiation, and function (Nakayama et al., 2015; Nakayama et al., 2016; Nakayama et al., 2018; Nakayama et al., 2019; Patel et al., 2019; Patel et al., 2020; Jin et al., 2021a). In a study by Patel et al., dECM and polycaprolactone (PCL) were electrospun together to mimic the topographical architecture of native muscle with a fiber diameter range of 500–2000 nm (Patel et al., 2019). The hybrid scaffolds were capable of supporting myogenic cell growth and the secretion of myokine growth factors. The nanofiber scaffold promoted *in vivo* muscle regeneration in a murine model of gastrocnemius-soleus VML with increased myofiber (MHC^+^) regeneration and elevated activity of anti-inflammatory M2 macrophage (arginase^+^) compared to the control groups (Patel et al., 2020). Similarly, nano-scale topographies were used to guide myogenesis using elastomeric PDMS chips together with iMPCs to support mature myotubes *in vitro* and *de novo* muscle regeneration with induced functional restoration of injured quadriceps femoris muscles in a VML mouse model (Jin et al., 2021a).

Both micro- and nano-topographies have been shown to guide myogenesis and functional muscle regeneration *in vitro* and *in vivo* and the optimal topographical scale is dependent on several factors including the desired cellular interactions, type of cells, and substrate properties.

#### 4.2.3 How Skeletal Muscle dECM Topography Affects Cell Behavior- Porosity

The porosity of a biomaterial determines the rate of transportation of gases, removal of waste products, and diffusion of macromolecular proteins from the surrounding environment into the bulk space of the matrix (Choi et al., 2019; Jin et al., 2021a) (Figure 5F). Shapiro et al. demonstrated that different concentrations of SDS effect the pore size of the resulting dECM, such that increasing concentrations of SDS produced tissues with greater pore sizes (Shapiro et al., 2019) (Figures 5G,H). The larger pore sizes better supported cell infiltration and allowed for the controlled release of IGF-1 to enhance *in situ* muscle regeneration. In a study done by Jin et al., four different types of dECM-PCL substrates were thermally drawn into sheets or fibers following a salt leaching method to incorporate porosity (Jin et al., 2021b). The four types of substrates include non-porous sheets, porous sheets, non-porous fibers, and porous fibers. Culture of iMPCs on the four different substrates resulted greater expression of myogenic genes (MyoD1, Mrf5, Myog, ITGB1, ITGA7, and CDH2) within the porous group when compared to the non-porous group. Others have demonstrated better cell viability when using porous dECM scaffolds compared to non-porous hydrogels (Choi et al., 2019), implicating possible cell hypoxia when scaffolds are not sufficiently porous.

Combinatorial effects of alignment plus porosity have also been associated with improved cellular outcomes. In a comparison of aligned (pore size of 3.39 µm) versus randomly-oriented (pore size of 2.58 µm) muscle constructs, greater cell viability if iMPCs was seen on the aligned scaffolds, which contained the larger pores, compared to the randomly-patterned scaffolds, which contained the comparatively smaller pore size (Jin et al., 2021a). Together, these studies underscore the significance and modulatory role of porosity in the use of skeletal muscle dECM to fabricate regenerative biomaterials; excessively high porosity will lead to lower structural integrity (Jakus et al., 2018) and a lack of porosity will potentiate hypoxia and cell death (Jin et al., 2021a).

## 5 Biophysical Properties: Mechanical Properties

Cell biomechanics are constantly at play in the regulation of tissue homeostasis and response to injury. In native tissues, cells are naturally exposed to physical forces such as compressive loading, tensile strain, shear, and electrical stimulation (Rando and Ambrosio, 2018). These dynamic mechanical cues transduce signals from the surrounding extracellular environment through the cell membrane and into the cell’s nucleus to influence the transcriptional programs that drive morphogenesis. By modulating the process of mechanotransduction, ECM-derived biomaterials can guide cells towards tissue remodeling, regeneration, and ultimately repair following injury.

### 5.1 Cardiac dECM Mechanical Properties

Disease, injury, and aging cause irreversible remodeling to cardiac ECM leading to fibrosis and a subsequent increase in stiffness, decreased contractility, and eventual heart failure. Due to the dynamic loading and contractile demands of the heart, an effective engineered therapeutic must support and recapitulate these functional mechanical properties. While cardiac dECM has been used to enhance the mechanical integrity of engineered cardiac tissues, the exact contribution of specific dECM biomechanical properties such as architecture, elasticity, and stiffness to the overall outcomes is not well-established (Silva et al., 2016).

#### 5.1.1 How Different Decellularization Protocols Affect Cardiac dECM Mechanical Properties

The exact relationship between specific mechanical properties and the decellularization protocols used to generate these cardiac dECMs is not well-characterized. While there is a consensus that restoration of functional cardiac mechanics is a key benchmark of a successful regenerative technology, there is a marked lack of published studies that characterize the dECM mechanics (Behmer Hansen et al., 2021). However, some broad correlations can be made between some decellularization approaches and the resulting effects on cardiac dECM elasticity and strength. Decellularization protocols that utilize SDS and Triton X-100 for long periods of time risk damaging collagen and elastin fibers by decreasing fiber thickness and losing ultrastructure (Merna et al., 2013; Gilpin and Yang, 2017; Lee et al., 2017; Behmer Hansen et al., 2021). These alterations to ECM glycoproteins can have detrimental effects as they play a role in maintaining cardiac elasticity and tensile strength (Behmer Hansen et al., 2021). Merna et al. measured compressive modulus following various whole heart perfusion decellularization protocols. They found decellularization with triton increased the compressive modulus by 150% in comparison to the native heart while a trypsin plus triton regimen decreased the compressive modulus by <20% (Merna et al., 2013). Careful preservation of dECM mechanical properties and elasticity is especially challenging for perfusion systems which risk overinflating the heart and causing damage to the elastin fibers. Therefore, methods have been developed that optimize perfusion and gravitational forces to minimize mechanical strain during decellularization. Lee et al. found that inverting the heart at a -45° angle, improved perfusion efficiency and also improved retention of ECM proteins and heart shape (Lee et al., 2017).

#### 5.1.2 How Cardiac dECM Mechanical Properties Affect Cell Behavior

Decellularized cardiac ECM provides mechanical cues that have the potential to improve cell attachment, differentiation, and maturation (Lu et al., 2013; Williams et al., 2014; Fong et al., 2016; Sarig et al., 2016; Das et al., 2019; KC et al., 2019; Hochman-Mendez et al., 2020; Mesquita et al., 2021). Specifically, cardiac dECM substrate stiffness can guide cell migration, proliferation, differentiation, and maturation (Merna et al., 2013; Perea-Gil et al., 2018). It has been shown that cardiomyocytes prefer softer substrates; when cultured on poly(ethylene glycol) and fibrinogen (PEGylated fibrinogen) hydrogels, an inverse correlation was found between material stiffness and the magnitude of cardiomyocyte contraction (Shapira-Schweitzer and Seliktar, 2007). As a therapeutic, dECM can provide mechanical support to the injured myocardium and must closely match the native contractile properties. If the dECM does not reflect native stiffness or the recellularized dECM construct contains immature cells, arrhythmias and a cascade of negative immune responses will occur leading to further scarring (Zhang et al., 2018). Therefore, dECM scaffolds must recapitulate the stiffness and mechanical properties of native myocardium as well as support cardiomyocyte contraction and maturation.

### 5.2 Skeletal Muscle dECM Mechanical Properties

Much like cardiac dECM, the influence of skeletal muscle dECM mechanical properties on myogenesis and muscle regeneration is not well-characterized. The biophysical dECM properties such as biochemical composition and topographical patterning are often the primary targets for modulation of cell behavior and less attention is given to controlling cellular interactions via classical tissue biomechanics. However, replicating the mechanical properties of skeletal muscle are a crucial component in the design of engineered skeletal muscle and regeneration *in vivo* (Lacraz et al., 2015).

The compressive stiffness of skeletal muscle dECM is heavily influenced by the decellularization reagents. In a study by Reyna et al. that compared three different decellularization methods, it was found that the SDS/Triton X and Triton X-only treated tissues had similar stiffnesses to the collagen control, while a LatB treatment produced the least stiff tissue across all groups (Reyna et al., 2020). While this specific study did not examine the downstream effects of stiffness on cellular interactions, two other studies found that the specific dECM that most closely resembled the stiffness of native muscle, promoted the greatest myogenesis *in vitro* and muscle regeneration *in vivo* (Choi et al., 2016; Jin et al., 2021b). To mimic the native structure of muscle, Choi et al. 3D printed muscle scaffolds using dECM bioinks (Choi et al., 2016). The dECM printed muscle scaffold exhibited a stiffness (12 ± 3 kPa) similar to that of native muscle (≈12 kPa) and also supported myoblast proliferation, myotube formation, and myogenic differentiation *in vitro*.

Mechanical variance is unavoidably introduced when dECM is fabricated into scaffolds with varying physical properties, and likely plays a contributing role in guiding subsequent cellular interactions. Jin et al. fabricated two types of muscle constructs by thermal drawing; a porous/fibrous scaffold (12.4 ± 3.5 kPa), and a porous/sheet-like scaffold (29.0 ± 3.8 kPa) (Jin et al., 2021b). The porous/fibrous scaffold had a stiffness that was most similar to native muscle (11.5 ± 1.3 kPa) and outperformed all other groups in myogenesis both *in vitro* and *in vivo*. Similar to cardiac dECM, the regenerative potential of skeletal muscle dECM is optimized when the scaffold’s mechanical properties most closely resemble that of the native tissue.

## 6 Emerging Research

The complementary fields of cardiac and skeletal muscle dECM research will continue to evolve and pioneer novel methods and tools for regenerative medicine. There are some very recent studies that stand apart in novelty and provide valuable insights and first steps that have the potential to take the field in a new direction. Herein, select studies are described that approach dECM research from an unexpected direction. While many of these studies were published within the past few years, they describe a dECM application that is a drastic departure from where the majority of the field currently resides.

### 6.1 Emerging Research in Cardiac dECM

The initial translational goal for cardiac dECM research was to use whole decellularized hearts as a therapeutic alternative for waitlisted organ transplant candidates. While this approach has yet to meet the clinical milestones necessary for a patient-applied treatment, cardiac dECM is nevertheless a versatile and powerful tool used in cardiomyocyte differentiation, maturation, and proliferation (Hochman-Mendez et al., 2020; Mesquita et al., 2021). It has also shown great promise as a therapeutic for the treatment of myocardial infarction, either in the form of a solubilized injectable gel or as an intact cardiac patch. (Sarig et al., 2016; Wang et al., 2016; Wang et al., 2019; Wang et al., 2020). However, despite the many studies demonstrating the utility of dECM for cardiac applications, there remains a gap in knowledge and bottlenecks in clinical translation.

To tackle the ongoing challenge of obtaining a renewable and robust supply of human cardiac dECM, one group has looked towards the use of cardiac organoids as a cultured source for dECM. Chiang et al. used organoids derived from human umbilical cord blood mesenchymal stem cells to lay down a structural protein niche that was then decellularized to provide a dECM scaffold (Chiang et al., 2021). The resulting dECM was shown to possess both essential microarchitecture and functional bioactivity (Chiang et al., 2021). This approach can be easily scaled up to produce large quantities of tissue-specific human ECM under reproducible and controlled laboratory conditions. Through the use of organoid-derived dECM, several major bottlenecks are circumvented by moving away from highly variable animal-derived products towards an approach that could be more easily standardized in a higher throughput setting.

Another approach for dECM cardiac tissue engineering that circumvents the need for healthy cardiac tissue as source material, is the use of other organs such as placenta or small intestine submucosa (SIS) for cardiac applications. Jiang et al. decellularized rat placentas which are highly vascularized, rich in ECM, and more readily available than hearts (Jiang et al., 2021). They recellularized the placenta with hiPSC-CMs and observed spontaneous contraction at a rate of 40—100 bpm. The hiPSC-CMs were also responsive to isoprenaline and had synchronized electromechanical propagation with external electrical stimulation. Additionally, the decellularized placenta significantly upregulated mRNA expression of genes related to cardiomyocyte structure, maturation, and conduction compared to cells culture on Matrigel coated tissue culture plastic. To test the therapeutic efficacy of their bioengineered cardiac patch (BCP), a rat myocardial infarction model was used. BCPs significantly improved left ventricular function, decreased infarct size, and increased cell retention compared to decellularized placenta or hiPSC-CMs alone after 4 weeks (Jiang et al., 2021).

The general concept of repurposing non-cardiac dECM for cardiac applications has also garnered significant interest and excitement with the emerging field of plant decellularization. Although decellularized vegetal scaffolds do not contain identical ECM components to decellularized cardiac tissues, their use has increased in popularity in recent years due to their innate networks for fluid transportation that resembles the human tissue vasculature. Additionally, plant tissue possesses similar mechanical properties, are highly accessible at a low cost, and non-immunogenic in nature (Harris et al., 2021). Plant tissue is primarily composed of cellulose, a well-studied, biodegradable compound that is widely used in a variety of clinical applications including wound dressing and drug delivery (Harris et al., 2021). Decellularized vegetal scaffolds are often coated with fibronectin, collagen, or poly-l-lysine (PLL) to promote cellular attachment and tune mechanical properties (Robbins et al., 2020; Harris et al., 2021). For cardiac tissue engineering, decellularized spinach (*spinacia oleracea*) and tomato (*solanum lycopersicum*) leaves have been identified as the ideal source material because their mechanical properties are in the range of human cardiac tissue (Gershlak et al., 2017; Robbins et al., 2020; Harris et al., 2021). Gershlak et al. decellularized spinach leaves, coated with fibronectin, and recellularized the vascular component with human umbilical vein endothelial cells (HUVECs) and the surface of the leaves with a combination of human mesenchymal stem cells (hMSCs) and hiPSC-CMs. The vasculature remained patent and perfusable and all cell types adhered and matured over the course of the 21 days study. The hiPSC-CMs began to spontaneously contact 5 days after seeding, exhibiting a 1% contractile strain and a contractile rate of 0.8 Hz (Gershlak et al., 2017). More recently, the same group recorded 3.94 ± 1.20% contractile strain which they hypothesized was due to their updated decellularization protocol which uses 1% SDS instead of 10% SDS (Robbins et al., 2020). While these results are promising, the contractile strain is still much smaller than the of adult myocardium (15.9–22.1%) (Yingchoncharoen et al., 2013). Therefore, significant work is still needed to meet the clinical milestones necessary for this cardiac patch to become a patient-applied treatment. Nonetheless, the use of plant-derived dECM for regenerative cardiac applications is an exciting new direction and may catalyze a paradigm shift and permenant new direction for cardiac decellularization.

### 6.2 Emerging Research in Skeletal Muscle dECM

The gold standard technology in skeletal muscle decellularization is experiencing a gradual shift away from the use of whole acellular tissue scaffolds towards more controlled 3D bioprinted approaches. Skeletal muscle dECM is now often solubilized into a bioink for extrusion printing, which allows for the construction of complex geometries and patterns that can be printed in multiple layers to support different cell types and deep vascular networks. A major advantage of muscle dECM printing is the ability to mass produce large quantities of bioactive materials, which has the potential to increase accessibility and thereby pave a smoother road towards clinical adoption as a therapeutic for muscle regeneration. However, despite these many advancements in skeletal muscle dECM 3D-printing, the ability to commercialize dECM using strictly bio-printing technology has yet to be fully realized (Dzobo et al., 2019). Many of the same bottlenecks originally associated with whole tissue decellularization, such as the yield of dECM, tissue supply, and cost, are carried over into 3D bioprinting approaches. These factors all still need to be evaluated before large-scale commercialization is possible. Therefore, similar to cardiac tissues, alternative sources of dECM materials have been a recent focus for skeletal muscle regeneration.

As an alternative to animal-derived proteins, some select plants are emerging as materials for engineering aligned muscle fibers. Monocotyledon plants, such as green onions, have a highly structured parallel architecture made up of cellulose. Decellularized monocotyledon plants possess anisotopy similar to striated human muscle tissue and were show to guide myogenesis through these topographical cues (Cheng et al., 2020). A wide variety of plants were decellularized using a basic protocol of 1% SDS for a 3 weeks duration. Out of the fruits and vegetables that were decellularized in the study, the monocotyledon plants such as asparagus, green onions, leeks, and celery all exhibited good anisotropic and aligned scaffold structures. This unidirectional structure in green onion plants, comprised of grooves that are about 20 µm wide and 10 µm deep, served as natural topographical templates for myogenesis (Cheng et al., 2020). Specifically, decellularized plants scaffolds seeded with human skeletal muscle cells (hMSCs) and C2C12 myoblasts induced a higher level of anisotropic organization of both cell types when compared to glass coverslip controls. Furthermore, the plant scaffolds demonstrated greater myogenesis in the form of myotube formation compared to controls. In the cardiac study by Gershlak et al., which also utilized dicotyledon plants (decellularized spinach leaves), it was shown that a wide range of cell types including endothelial cells and mesenchymal stem cells, are supported by these decellularized plant materials (Gershlak et al., 2017). Given the diverse assortment of decellularizable plant structures, this approach reduces the potential production cost, impact on the environment, and offers a vegan-friendly foundation for bringing this technology to mass production.

Engineering plant-based solutions could also solve a major problem in the meat production industry. About 60% of global greenhouse gas emissions can be traced back to the production of animal-based foods (Xu et al., 2021). Bioartificial lab-grown meat is an emerging area in the biotechnology sector and has the potential to provide edible meat alternatives to large sectors of the global population. There are currently over 70 cultured meat startup companies and who collectively raised $366 million last year (Byrne and Murray, 2021). These companies produce cell-based meat that mimics beef, poultry, pork, and seafood and many base their products on derivatives of dECM. In a specific study by Jones et al., decellularized spinach leaves were used as a matrix for culturing bovine satellite cells as they were developed into edible “meat” (Jones et al., 2021). The decellularization of spinach leaves utilized a 1% SDS protocol, and resulted in a dECM structure filled with a deep hollow vascular network suitable for cellular infiltration. Approximately 25% of the bovine satellite cells stained positive for myosin heavy-chain expression and showed good cytoskeletal alignment (average kappa value of 0.71 ± 0.1) supportive of anisotropic myogenesis. While the field of cultured meats is still relatively young, if successful, this industry has the potential to make a massive impact on the supply chain of food worldwide.

## 7 Commentary on Future Directions for Cardiac and Skeletal Muscle dECM

Although the use of dECM in tissue engineering has led to many paradigm-shifting insights, there is still much work to be done before it can become standard clinical practice. There needs to be better methods for standardization of decellularization protocols and dECM characterization following decellularization. Many of the published protocols do not specify durations and often assess decellularization visually based on non-quantitative color-based metrics. To overcome batch-to-batch variations, decellularization protocols should be followed by characterization of both the biochemical and mechanical properties of the dECM using such tools as liquid chromatography-mass spectrometry, atomic force microscopy, and electron microscopy. It is crucial to retain the structure and bioactive components of the dECM and it has been shown that overexposure to many of the decellularization agents cause irreversible damage. Conversely, incomplete decellularization results in unwanted immune responses and limits recellularization. To date, there is no universal decellularization protocol as there are many complex factors that influence how a tissue will decellularize. Oftentimes, the selected protocol is a true compromise built on the sacrifice of some biophysical properties in favor of others. As our understanding of the underlying mechanisms driving cell-dECM interactions are uncovered, the fields of cardiac and skeletal muscle decellularization will evolve and refine to increase in translational relevance and expand in impact on human health.
